# Understanding Transcription Factor Regulation by Integrating Gene Expression and DNase I Hypersensitive Sites

**DOI:** 10.1155/2015/757530

**Published:** 2015-09-03

**Authors:** Guohua Wang, Fang Wang, Qian Huang, Yu Li, Yunlong Liu, Yadong Wang

**Affiliations:** ^1^School of Computer Science and Technology, Harbin Institute of Technology, Harbin, Heilongjiang 150001, China; ^2^Instrument Science and Technology, Harbin Institute of Technology, Harbin, Heilongjiang 150001, China; ^3^School of Life Science and Technology, Harbin Institute of Technology, Harbin, Heilongjiang 150001, China; ^4^Department of Medical and Molecular Genetics, Indiana University School of Medicine, Indianapolis, IN 46202, USA; ^5^Center for Computational Biology and Bioinformatics, Indiana University School of Medicine, Indianapolis, IN 46202, USA

## Abstract

Transcription factors are proteins that bind to DNA sequences to regulate gene transcription. The transcription factor binding sites are short DNA sequences (5–20 bp long) specifically bound by one or more transcription factors. The identification of transcription factor binding sites and prediction of their function continue to be challenging problems in computational biology. In this study, by integrating the DNase I hypersensitive sites with known position weight matrices in the TRANSFAC database, the transcription factor binding sites in gene regulatory region are identified. Based on the global gene expression patterns in cervical cancer HeLaS3 cell and HelaS3-ifn*α*4h cell (interferon treatment on HeLaS3 cell for 4 hours), we present a model-based computational approach to predict a set of transcription factors that potentially cause such differential gene expression. Significantly, 6 out 10 predicted functional factors, including IRF, IRF-2, IRF-9, IRF-1 and IRF-3, ICSBP, belong to interferon regulatory factor family and upregulate the gene expression levels responding to the interferon treatment. Another factor, ISGF-3, is also a transcriptional activator induced by interferon alpha. Using the different transcription factor binding sites selected criteria, the prediction result of our model is consistent. Our model demonstrated the potential to computationally identify the functional transcription factors in gene regulation.

## 1. Introduction

In molecular biology and genetics, transcription factors (TFs) are proteins that bind to DNA sequences specifically, thereby regulating the transcription of genetic information from DNA to messenger RNA [1]. Once bound to DNA, these proteins can promote or block the recruitment of RNA polymerase to specific genes, making genes more or less active. Transcription factors are essential for the regulation of gene expression. Under the effect of transcription factors, the various cells of the body can function differently though they have the same genome. Transcription factors bind to one or more sequence sites, which are called transcription factor binding sites (TFBSs), attaching to specific DNA sequences of the genes they regulate [2]. Transcription factor binding sites can be defined as short DNA sequences (5–20 bp long) specifically bound by one or more transcription factors [3]. The transcription regulation is carried out by the interplay between transcription factors and their binding sites in DNA sequences; thus the prediction of TFBS is a vital step to understand the mechanism of transcription regulation and construct the network of transcription regulation. With the development of DNA microarrays and fast sequencing technique, many transcription factor binding sites have been identified by using experimental methods such as ChIP-chip and ChIP-Seq [4–6]. Because these methods will consume many experiment materials and many TFs have no corresponding antibodies, biological experimental methods cannot identify all TFs in the genome. Hence, many different computational methods have been proposed to search for additional members of a known transcription factor binding motif or discover novel transcription factor binding motifs.

In recent years, many computational methods such as regression based approaches have been proposed to discover transcription factor binding sites based on gene expression data. These methods can model the relationship between gene expression and transcription factor binding motifs in the promoter regions [7–9]. Bussemaker et al. proposed a simple linear model between gene expression and transcription factors using the TFBSs counts in the promoter region [10]. Based on this model, instead of the counts of TFBSs, Conlon et al. used position weight matrices (PWMs) to identify the motif candidates on upstream of genes [11]. In these previous methods, the whole promoter regions were always used as transcriptional regulatory regions that include TFBSs. As we all know, promoter regions are much longer than TFBSs; therefore, it will be better for TFBS prediction if we can narrow down the potential transcription factor binding region.

As early as the 1980s, the gene transcription was found to be related with the sensibility to DNase I (deoxyribonuclease I) of chromatin [12]. The sensibility to DNase I of chromatin which contains the actively transcribed genes is 100 times stronger than the one of the chromatin which does not contain the actively transcribed genes [13]. In 2013, Sheffield et al. [14] found that TFBSs were correlated with the DNase I hypersensitive (DHS) sites. The structure of the chromatin that contains DHS sites is looser, so that gene regulatory proteins can bind to these regions preferentially to exert biological functions [15–18]. Within the DHS sites, the regions are not digested easily and protected by specific proteins which probably are gene regulatory proteins such as transcription factors. In this study, the DHS sites were combined with gene expression data to deduce the target genes, and it was found that approximately 71 percent of DHS sites associated with at least one gene and some of these DHS sites associated with up to 44 genes, and among these genes the protein-coding genes were more than RNA genes. Using Encode ChIP-Seq data, the transcription factor binding sites were compared to the DHS sites, which showed highly overlapping percentage. Hence, the DHS sites in the promoter region can be used to identify TFBSs [19].

In our previous study, a model-based procedure has been developed to predict the functional TFBSs. The model utilized known position weight matrix to identify potential TFBSs in the gene promoter regions and built quantitative relationship between the TFBSs and gene expression levels. The transcriptional regulatory region was arbitrarily defined as the upstream region of transcription start site. In this study, we proposed a modified method that combined the DNase I hypersensitive sites with promoter regions to promote the accuracy of TFBS identification and recognize the regulatory function of transcription factors.

## 2. Methods

### 2.1. Biological Model System

The cervical cancer HeLaS3 cell, which is a clonal derivative of the parent HeLa cell, has been very useful in the clonal analysis of mammalian cell populations relating to chromosomal variation, cell nutrition, and plaque-forming ability. In recent years, as a tier of 2 cell types of ENCODE project, large sets of genome-wide study used the next generation sequencing technology to investigate gene expression, transcription factor binding sites, histone modification, and DNase I hypersensitive sites in HeLaS3 cell line. In this study, using genome-wide gene expression profile combined with DNase I hypersensitivity data, we developed a new method to predict the most important transcript factor in interferon alpha treated HeLaS3 cell line.

### 2.2. Gene Expression and DNase I Data Set

The gene expression profiles of HeLaS3 and HeLaS3 treated by interferon alpha for 4 hours were downloaded from Gene Expression Omnibus Database (GEO number: GSE15805), where Affymetrix Human Exon 1.0 ST Array was used to access the global gene expression patterns in 3 and 2 replicates. The DNase I data set of HeLaS3 used in this study was freely available for downloading from the uniform DNase I HS track of UCSC NCBI37/hg19 ENCODE (http://genome.ucsc.edu/encode/).

### 2.3. Differential Expressed Gene Identification

Each gene expression array of 3 HeLaS3 replicates and 2 HelaS3-ifn*α*4h replicates has been done the RMA normalization used Affymetrix Power Tools (APT) and removed the batch effects using ComBat in the previous study [20]. We utilized the Quantile Normalization [21] to eliminate the difference among the parallel experiments and then used the Scaling Normalization [22] to eliminate the difference between two cell types. The genes not reliably detected in at least one of the two cells were removed and only the protein-coding genes were picked up. After *t*-test calculation, we selected 197 probe sets by *P* < 0.05 and fold change >  ±2; the expression levels of them were altered significantly. Removing the probe sets that were not reliably detected and that had absent annotation; finally, 181 differentially expressed genes [23] were left for analysis, in which 121 were upregulated and 60 were downregulated.

### 2.4. TFBS Prediction in DHS Sites

For the 181 differentially expressed genes, the DHS sites which located in the 1,000 bp upstream and 500 bp downstream of transcription start sites were picked up as transcriptional regulatory regions. Human RefSeq transcript annotation (hg19 genome assembly) and regulatory sequence were retrieved from the UCSC Genome Browser. 2188 position weight matrices (PWMs) in the TRANSFAC database were used to predict the transcription factor target genes. For each TF-DHS pair, the similarity scores were calculated by scanning the PWM of the transcription factor along the sequence of DHS site and the maximum score was selected as the binding affinity between the transcription factor and DHS site. For each PWM, we selected top 5000 DHS sites with highest similarity scores in genome-wide as potential TFBS.

### 2.5. The Prediction of Functional Transcription Factor

In order to describe the correlation between the genes expression levels and the binding affinity of transcription factors in DHS sites, a simplified quantitative relationship is established using a linear model:

(1) $$\begin{array}{c} g_{k}=\sum_{i\in T_{k}}\left(\;\sum_{m}d\left[\;m,i\;\right]\;\right)x_{i}, \end{array}$$

where *g*_*k*_ is the logarithmic ratio of mRNA expression levels of the *k*th gene in the treatment group comparing to control group, *d*[*m*, *i*] is the matching score of *i*th PWM in the *m*th DHS sites within transcriptional regulatory region of the *k*th gene, *T*_*k*_ is the number of all the TFBSs having occurrences in the regulatory region of the *k*th gene, and *x*_*i*_ is the functional level of the *i*th PWM. The biological implication of this equation is that the measured gene expression level *g*_*k*_ is modeled by the effect of transcription, controlled by 5′* cis*-acting elements. Because the expression level of genes we used in this study was Log2 RMA expression value, *g*_*k*_ was calculated according to the following formulation:

(2) $$\begin{array}{c} g_{k}=s_{k,\text{Treatment}}-s_{k,\text{Control}}, \end{array}$$

where *S*_*k*,Treatment_ is the logarithmic ratio of mRNA expression levels of the *k*th gene in the treatment group (HelaS3-ifn*α*4h) and *S*_*k*,Control_ is the logarithmic ratio of mRNA expression levels of the *k*th gene in the control group (HelaS3).

The linear model only described the quantitative relationship between gene expression levels and PWMs of one differentially expressed gene. Thus, the model can be rewritten in a matrix formulation:

(3) ZCDX,X=CDTCD−1CDTZ,

where *Z* = (*g*_*k*_); *X* = (*x*_*i*_) and *C* is the marking matrix recording whether the DHS sites are within the transcriptional regulatory regions of differentially expressed genes or not. If the *j*th DHS site is within the transcriptional regulatory region of the *i*th gene, *C*[*i*, *j*] = 1; otherwise *C*[*i*, *j*] = 0. *D* is the score matrix representing the maximum score of each motif candidate in each DHS site. The model error based on a given selection of TFs will be defined as the sum square of the differences between observed and predicted mRNA expression levels:

(4) $$\begin{array}{c} e=\overset{n}{\underset{k=1}{\sum }}{\left(\;g_{k}-\sum_{i\in T_{k}}\left(\;\sum_{m}d\left[m,i\right]\;\right)x_{i}\;\right)}^{2}, \end{array}$$

where *e* is the error of this model and *n* is the total number of differentially expressed genes. This equation can be rewritten in a matrix formulation:

(5) ErrZ−CDX=Z−CDCDTCD−1CDTZ.

In this study, we iteratively computed the model error of each PWM for *N*_*p*_ = 100,000,000 times. In each iteration, the program selected *n*_*t*_ = 5 PWM candidates randomly. The model error of each set of PWMs was calculated. Meanwhile, we assigned a score value, transcription factor's contribution value (TFCV), for each PWM candidate. The TFCV can be calculated by the following formulation:

(6) $$\begin{array}{c} \text{TFCV}=\sum_{N}\frac{1}{{\text{Err}}^{2}}, \end{array}$$

where Err is the model error and *N* is the number of selected PWM candidates in each iteration. If Err is smaller, namely, TFVC score is higher, the transcriptional function of PWM corresponding transcription factor will be more significant. Meanwhile, the cumulative TFs' functional levels (TFL) were calculated by the sum of *x*.

The program of functional transcription factor prediction can be summarized as follows.

(1) Calculate the matrix *Z* of expression levels of all the genes in the HelaS3-ifn*α*4h comparing to the HelaS3.
(2) Extract the DNA sequences of DHS sites of HelaS3 and calculate the score matrix *D* using PWM. For each PWM, the threshold value (ts) is set as the 5000th highest score.
(3) Construct the matrix *C* by comparing the position of DHS site and gene's regulatory region coordinate in the genome.
(4) Randomly pick *n*_*t*_ PWMs from all 2188 PWM candidates.
(5) Calculate the predicted model error Err.
(6) Calculate the TFCV and TFL of each PWM which is randomly picked in this iteration.
(7) Add the current transcriptional contribution score to the cumulative TFs' contribution value (TFCV) and add the current function level to the cumulative TFs' functional levels (TFL).
(8) Repeat the program (4–7) *N*_*p*_ times.

## 3. Results

### 3.1. Overlapping between DHS Sites and TFBS of HelaS3

**Figure 1:**
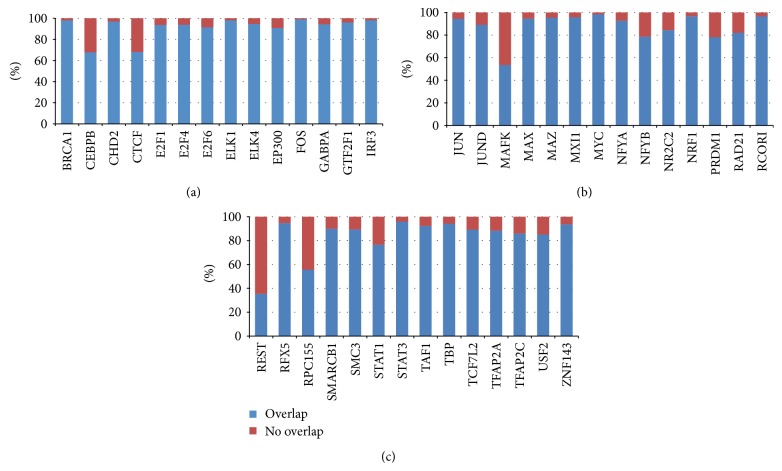
Overlapping between transcription factors binding regions and DHS sites. The blue bar and red bar represent the percentage of transcription factors that overlap and do not overlap with the DNase I hypersensitive sites, respectively.

The transcription factors ChIP-Seq data [16, 17] and DNase I hypersensitivity sites of HelaS3 cells were downloaded from the UCSC Genome Browser. After filtering out the ChIP-Seq experiments with poor quality, 42 TFBS profiles were considered the overlapping analysis with DHS sites in HelaS3 cells (Figure 1). Notably, we found that the binding sites of 26 transcription factors had more than 90% overlap and only 5 factors had less than 70% overlap with DHS sites. Among these 5 factors, CTCF which often acts as a chromatin “insulator” creates boundaries between topologically associating domains in chromosomes. Therefore, transcription factors tend to bind to the DHS sites and we can utilize the DHS sites to improve the accuracy of transcription factor binding sites prediction.

### 3.2. Functional Transcription Factor Identification

**Figure 2:**
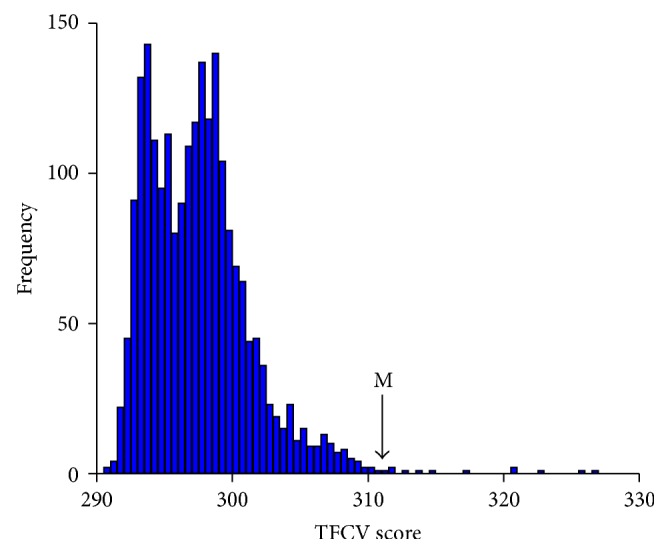
The histogram of TFCV scores for 2182 known PWMs. The *x*-axis is TFCV score and the *y*-axis is the frequency of the occurrence of TFCV for all known PWM.

Potential PWMs which corresponded to the binding sequence of a specific transcription factor were selected based on the binding affinity within DHS sites in the gene promoter region, as detailed in the methods. In order to predict the transcription factor binding sites, we calculated the score matrix *D* which stored the maximum scores as the binding affinity between the transcription factors and DHS sites. For each PWM, we selected top 5,000 matching positions with the highest similarity scores in the DHS sites genome-wide as potential TFBSs. After calculating our model iteratively, potential PWMs were selected based on the TFCVs of all PWM candidates. The histogram of TFCVs score of PWMs candidates is shown in Figure 2. In these PWM candidates, not all of them are real functional transcription factor binding sites. According to the methods, if the TFCV scores of PWMs are higher, their contributions to the alteration of gene expression are more significant. We selected the top 10 PWMs with the highest TFCV scores. The TFCV scores and the TFL values of these 10 PWM candidates are shown in Table 1. Significantly, 6 out 10 PWMs, including IRF, IRF-2, IRF-9, IRF-1, and IRF-3, ICSBP, belong to interferon regulatory factor family and upregulate the gene expression levels responding to the interferon treatment. ISGF-3 is also a transcriptional activator induced by interferon alpha. Among 10 PWMs, 9 received positive TFL values. This implies the increased capability of the 5′-end promoters in initiating transcription after treatment with interferon alpha.

### 3.3. Comparison of the Different TFBS Selection

**Figure 3:**
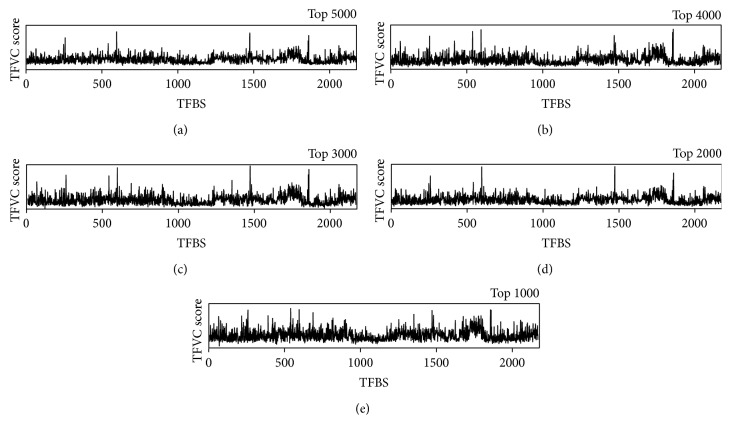
TFCV profile of 5 selected highest TFBS candidate models. The spectra of TFCV of all the PWMs while the threshold of potential TFBS is the 5000th, 4000th, 3000th, 2000th, or 1000th highest similarity score for each PWM. The *x*-axis corresponds to 2188 PWMs and the *y*-axis corresponds to TFCV scores.

**Figure 4:**
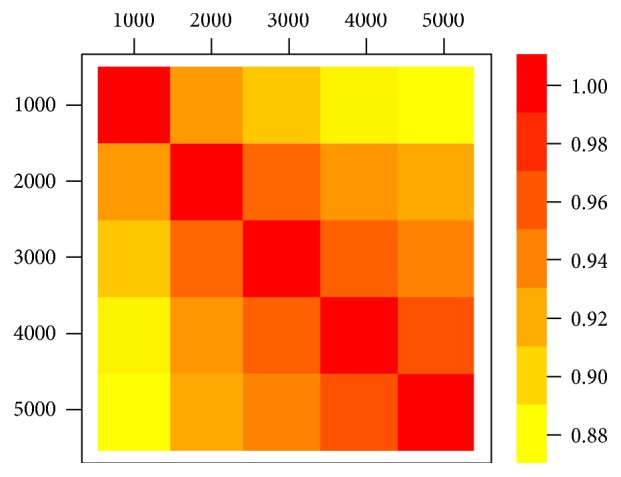
The cross-correlation coefficients of TFCV score among 5 selected highest TFBS candidate models.

To verify the accuracy of our model, we repeatedly run our model by changing the number of TFBSs to top 1000, 2000, 3000, or 4000 highest scores for each PWM. The TFCV profiles of each repeat computation are shown in Figure 3. We found that the distributions of TFCVs of all the PWM candidates in these 5 results were very similar. The Pearson correlation coefficient between the TFCV scores of each pair of predicted results was calculated. A heatmap corresponding to the Pearson correlation coefficient is shown in Figure 4. Obviously, the correlation between the prediction of top 1000 and top 5000 is the lowest (0.88), and the correlation between the prediction of top 4000 and top 5000 is the highest (0.96). The top 10 predicted PWMs with the highest TFCV score in all 5 calculations are shown in Table 2. Most of the top 10 PWMs are the same among these five prediction results, and most of them belong to interferon regulatory factor family.

## 4. Discussion

In this study, we modified the previous procedure ModifModeler to identify functional transcription factors. In the previous procedure, the transcription factor binding regions were set as the promoter regions [24]. To improve the accuracy of the identification of transcription factor binding sites, we reduced the searching space of transcription factor binding regions. We have known that transcription factors tended to bind to DNase I hypersensitive sites; thus we combined the DNase I hypersensitive sites with promoter regions to construct a new model. In our model, using DHS sites within transcriptional regulatory region of each differentially expressed gene to replace all promoter regions, the binding regions of transcription factors were shortened and the accuracy of predicting transcription factor binding sites was improved. In this study, our model predicted some transcription factor binding sites whose functions differed as a result of interferon-*α* treatment.

Our modified model predicted that 9 of the top 10 transcription factors showed upregulatory effects on gene expression after interferon-*α* treatment which was clearly shown in Table 1. These predicted top 10 transcription factors with the largest TFCVs made significant contribution to the alteration of gene expression after interferon treatment. After being treated by interferon, some mechanisms of HelaS3-ifn*α*4h have changed compared with HelaS3 and some transcription factors responding to the interferon treatment have shown significant contribution to the alteration of gene expression. Obviously, most of the predicted TFs belong to interferon regulatory factor family, such as IRF-1, IRF-2, IRF-3, and IRF-9, ICSBP, and upregulate gene expression under interferon treatment [25–27]. Meanwhile a factor named interferon-stimulated response element (ISGF-3) also contributes to the alteration of gene expression significantly. It also indicates that our modified model can identify transcription factors which induced the gene expression change.

The identification of transcription factor binding sites is still a challenging and meaningful area. In the future, the identification of transcription factor binding sites will be very important and helpful for the understanding of the gene regulation mechanism [28]. Gene expression is regulated by many different elements synthetically. To predict different regulatory elements and understand their function, we also need to modify our model to adapt to various gene regulatory elements, such as microRNA and RNA binding proteins. In summary, focusing on the integration with DNase I hypersensitive sites allows high accuracy in our prediction procedure. As we all know, the identification of transcription factor binding sites can be used in clinic to find the change of regulatory elements in damaged or diseased cells and then help with the therapy of disease in the gene expression level [29]. We believe that our optimized method will contribute to an existing analytical network of gene expression.

## Figures and Tables

**Table 1:** Transcription factor's contribution value (TFCV) and estimated TFs' functional levels (TFL) of top 10 selected PWMs.

Index	ID	TF name	PWM description	TFCV	TFL
1	M00772	IRF	Interferon regulatory factor family	326.928	14830.189
2	M01882	IRF-2	Interferon regulatory factor 2	325.779	14680.555
3	M02771	IRF-9	Interferon regulatory factor 9	322.969	15127.858
4	M00258	ISGF-3	Interferon-stimulated response element	320.613	9914.496
5	M01881	IRF-1	Interferon regulatory factor 1	320.501	15363.707
6	M02767	IRF-3	Interferon regulatory factor 3	317.408	11305.011
7	M00699	ICSBP	Interferon consensus sequence-binding protein	314.717	7242.987
8	M00248	Oct-1	Octamer factor 1	313.642	6287.612
9	M01235	IPF1	Homeodomain-containing transactivator	310.253	6593.312
10	M01857	AP-2 alpha	Activating enhancer binding protein 2 alpha	309.403	−3725.557

**Table 2:** The top 10 transcription factors with the highest TFCV score in 5 selected highest TFBS candidate model.

Index	Top 1000	Top 2000	Top 3000	Top 4000	Top 5000
1	ICSBP	IRF-9	IRF-2	IRF-2	IRF
2	IRF	IRF	IRF	IRF	IRF-2
3	IRF-3	ICSBP	IRF-9	IRF-9	IRF-9
4	ISGF-3	IRF-3	IRF-1	ISGF-3	ISGF-3
5	IRF-9	IRF-2	IRF-3	IRF-1	IRF-1
6	IRF-1	ISGF-3	ISGF-3	IRF-3	IRF-3
7	IRF	IRF-1	ICSBP	ICSBP	ICSBP
8	EAR2	IRF-7	EAR2	Oct-1	Oct-1
9	IRF-5	IRF-1	IRF-1	IPF1	IPF1
10	RREB-1	EWSR1-FLI1	Lim1	AP-2 alpha	AP-2 alpha
